# The Hidden Culprit in a Massive Episode of Hematemesis: A Dieulafoy's Lesion

**DOI:** 10.7759/cureus.824

**Published:** 2016-10-10

**Authors:** Sameen Khalid, Aamer Abbass, Tiffanie Do, Divyanshu Malhotra, Melanie Albors-Mora

**Affiliations:** 1 Florida Hospital, Orlando; 2 College of Medicine, University of Central Florida College of Medicine

**Keywords:** dieulafoy's lesion, hematemesis, angiography

## Abstract

A Dieulafoy’s lesion is described as a tortuous, dilated aberrant submucosal vessel that can penetrate through the mucosa and rupture spontaneously, resulting in severe gastrointestinal bleeding. The lesion is most commonly found in the proximal stomach. Historically, it has had up to an 80% mortality rate because of its tendency to cause intermittent but severe bleeding and diagnostic challenges. We report a case of a young male with recurrent severe upper gastrointestinal bleeding with extensive prior investigations failing to reveal the source of bleeding. Computed tomography angiography of the abdomen correctly identified Dieulafoy’s lesion of the stomach, and it was subsequently confirmed and successfully treated with interventional radiology (IR)-guided mesenteric angiography and embolization.

## Introduction

Dieulafoy’s lesions are tortuous, dilated aberrant submucosal arteries that can erode through the mucosa and rupture spontaneously and result in severe gastrointestinal (GI) bleeding [1]. The lesions are most commonly found in the proximal stomach, along the lesser curvature within 6 cm of gastroesophageal junction [2]. There have also been cases reported in the esophagus, duodenum, small intestine, and colon[3-4]. They account for 1-2% of all GI bleeding [2-4] and 6% of all upper GI nonvariceal bleeding [2, 4]. They have up to an 80% mortality rate because of their tendency to cause intermittent but severe bleeding and diagnostic challenges [3]. We report a case of recurrent severe upper GI bleeding due to a Dieulafoy’s lesion of the stomach, which was seen on computed tomography angiography (CTA) of the abdomen and subsequently confirmed and successfully treated with interventional radiology (IR)-guided mesenteric angiography and embolization.

## Case presentation

**Figure 1:**
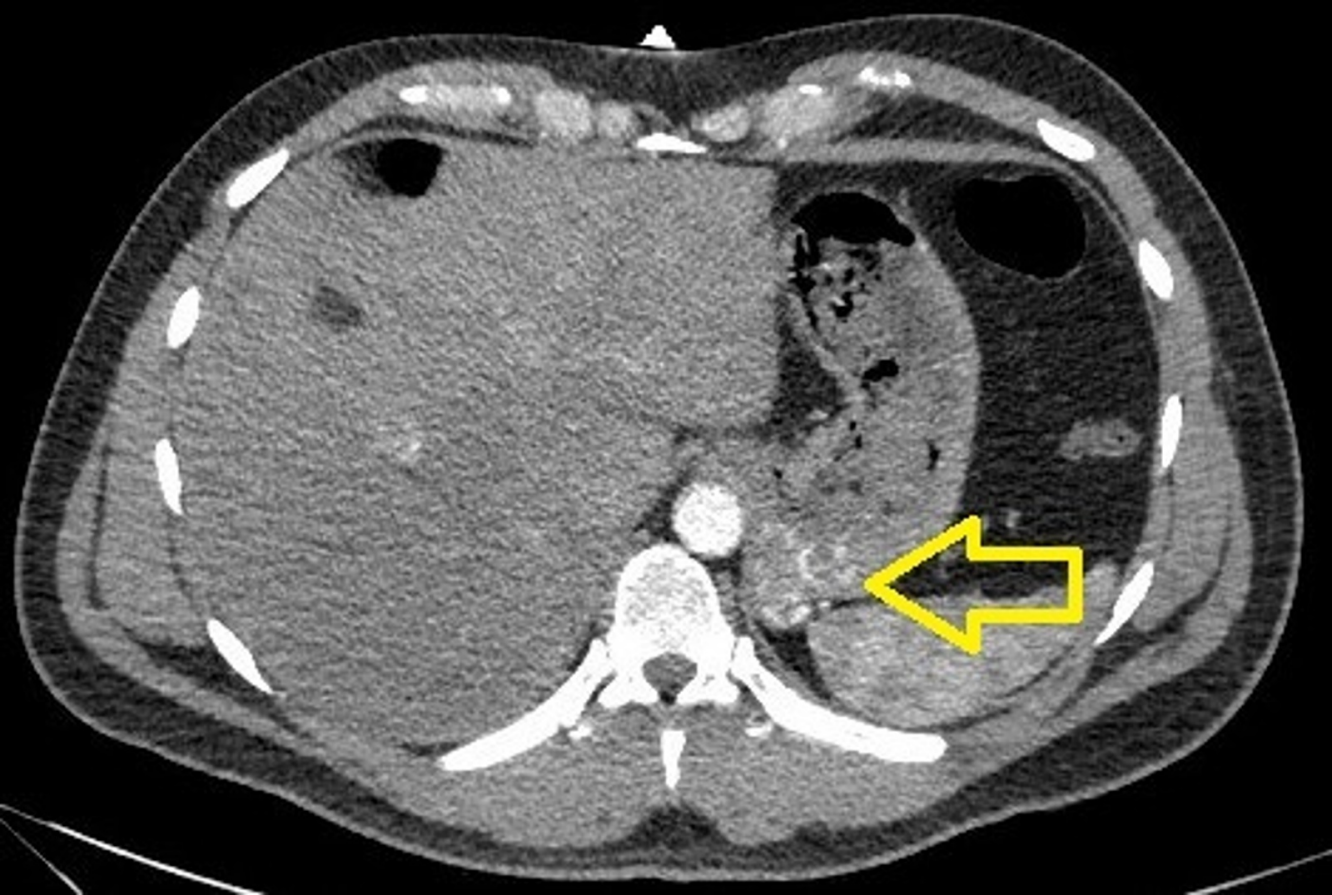
CTA of the abdomen showing a small tangle of enlarged tortuous blood vessels along the posterior cardia of the stomach.

**Figure 2:**
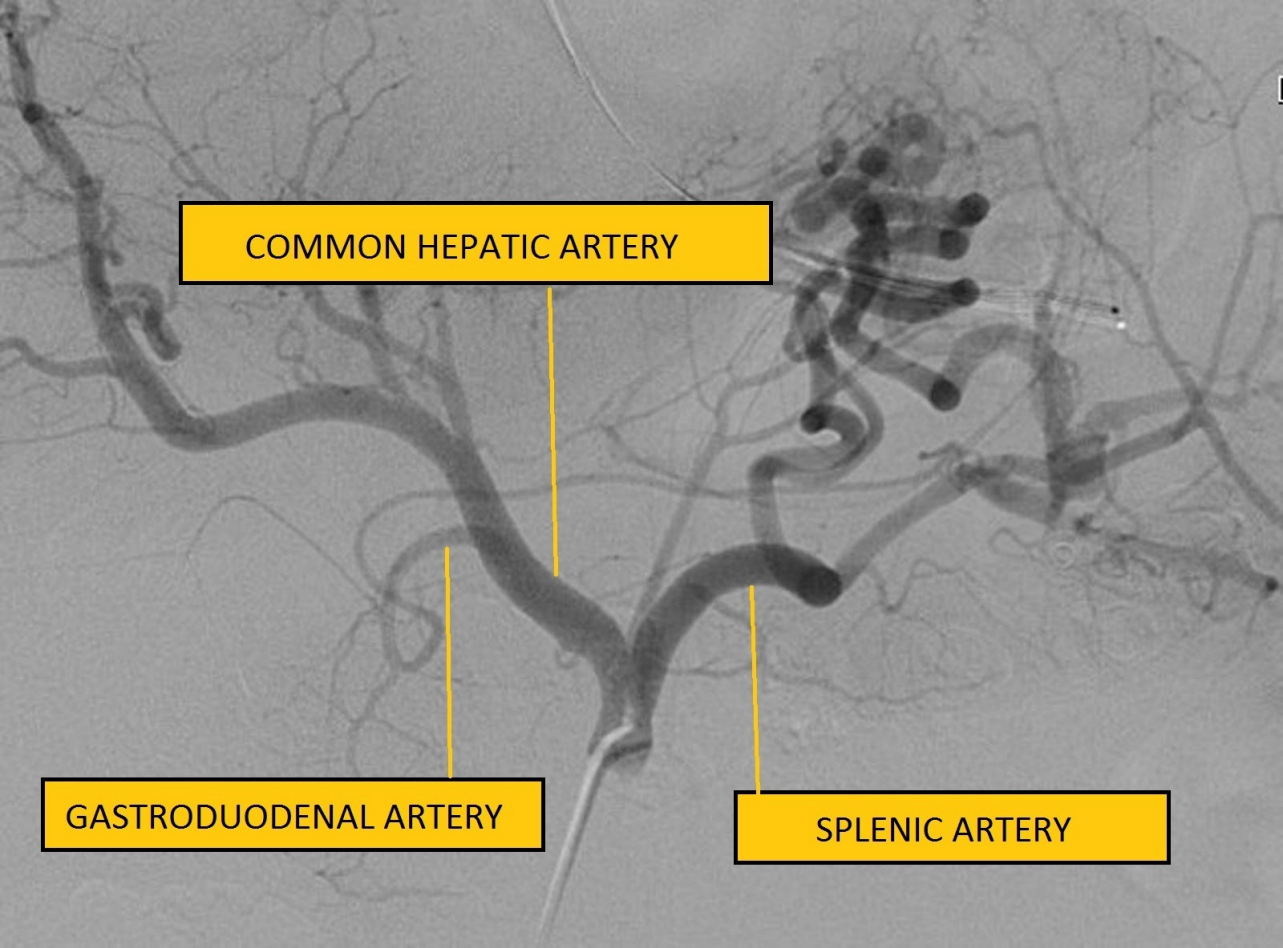
Celiac angiogram showing multiple hypertrophied, tortuous, non-tapering vessels arising from the proximal splenic artery.

**Figure 3:**
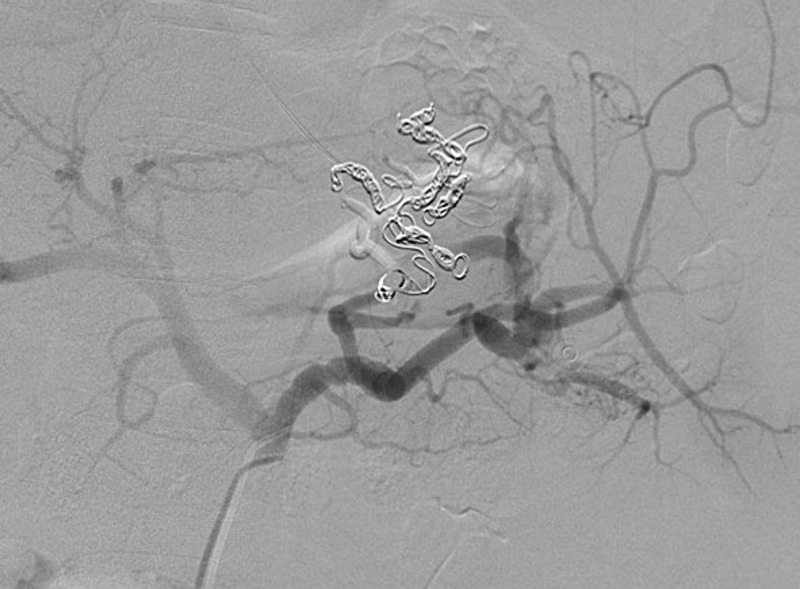
Successful coil embolization of multiple Dieulafoy’s lesions arising from the proximal splenic artery.

A 39-year-old Hispanic male presented with the sudden onset of melena and painless massive hematemesis. He denied use of tobacco and alcohol and had no history of nonsteroidal anti-inflammatory drug use. His past medical history was significant for gastroesophageal reflux disease and similar complaints of melena and hematemesis eight years previously. Extensive prior investigations, including computed tomography (CT) of the abdomen and pelvis, esophagogastroduodenoscopy (EGD), and nuclear medicine scan, failed to reveal the source of bleeding. Informed patient consent was obtained. He was hemodynamically stable at the time of presentation; however, his hemoglobin dropped from a baseline of 13 g/dl to 8 g/dl. The EGD showed a mild reflux esophagitis, acute duodenitis, and gastric varices in the fundus of the stomach. CTA of the abdomen showed a small tangle of abnormally enlarged tortuous bleeding vessels along the posterior cardia of the stomach with the absence of an early venous return, suggestive of multiple Dieulafoy’s lesions, as shown in Figure 1. An IR-guided mesenteric angiogram was subsequently performed with successful coil embolization of multiple Dieulafoy’s lesions arising from the proximal splenic artery and coursing through the region of the gastric cardia and fundus, as shown in Figures 2-3. The patient had an uneventful recovery and he was discharged home. He did not have any recurrence of bleeding, and his hemoglobin was stable at a three-month follow-up visit.

## Discussion

This case presented a classic example of hematemesis and illustrated the diagnostic challenge of identifying a Dieulafoy’s lesion in the absence of active bleeding. An extensive diagnostic evaluation was performed with laboratory tests, CT scan of the abdomen and pelvis, esophagogastroduodenoscopy, and nuclear medicine scan; however, the etiology was not found until CT angiography of the abdomen was performed, which suggested the presence of Dieulafoy’s lesion. A subsequent IR-guided mesenteric angiogram confirmed the diagnosis and provided means of treatment by embolization.

Bleeding episodes in patients with Dieulafoy’s lesions are usually self-limited but often intermittent and profuse [2]. Patients are often asymptomatic until they present with a massive upper gastrointestinal hemorrhage. Endoscopy is the diagnostic modality of choice to detect Dieulafoy’s lesion, which is seen as an active arterial pumping in an area without an associated mass or ulcer. However, due to the intermittent bleeding pattern of Dieulafoy's lesion, endoscopy often fails to provide an accurate diagnosis [2-4]. This is especially true in the absence of active bleeding as the surrounding mucosa is normal and there is no ulceration [3-4]. Endoscopy may also be unrevealing if the bleeding is brisk and blood pools up in the fundus of the stomach, obscuring the anatomy [3]. Dieulafoy’s lesions have also been known to be mistaken for other entities, such as arteriovenous malformations, aneurysms, Mallory-Weiss tears, and gastric varices, such as in our case, causing further diagnostic dilemma [3]. A recent review article suggested CT angiography as a diagnostic modality in cases where endoscopy fails to reveal the correct diagnosis [3]. Our case did support using CT angiography after failed endoscopy as it can recognize aberrant tortuous submucosal vessels. The lesions can then be confirmed and treated with minimally invasive angiography and selective arterial embolization [5]. The risk of rebleeding from Dieulafoy's lesions has been reported to range between 9-40% [4]; hence, there is a need to closely follow patients in the post-procedural period to monitor for potential rebleeding.

## Conclusions

The diagnosis of Dieulafoy’s lesions presents a formidable challenge for physicians and radiologists. Endoscopy is the diagnostic modality of choice, but it may be unrevealing due to the small size of the Dieulafoy’s lesion, normal appearance of surrounding mucosa, and intermittent bleeding patterns. Contrast-enhanced CT and angiography can be helpful in cases where initial endoscopy fails to reveal the diagnosis of Dieulafoy’s lesion and can offer minimally invasive therapeutic alternatives to surgical resection through selective arterial embolization.

## References

[1] Eloubeidi MA, El Majzoub NW (2012). Images in clinical medicine. Duodenal Dieulafoy's lesion. N Engl J Med.

[2] Shin HJ, Ju JS, Kim KD, Kim SW, Kang SH, Kang SH, Moon HS, Sung JK, Jeong HY (2015). Risk factors for Dieulafoy lesions in the upper gastrointestinal tract. Clin Endosc.

[3] Batouli A, Kazemi A, Hartman MS, Heller MT, Midian R, Lupetin AR (2015). Dieulafoy lesion: CT diagnosis of this lesser-known cause of gastrointestinal bleeding. Clin Radiol.

[4] Jeon HK, Kim GH (2015). Endoscopic management of Dieulafoy's lesion. Clin Endosc.

[5] Alomari AI, Fox V, Kamin D, Afzal A, Arnold R, Chaudry G (2013). Embolization of a bleeding Dieulafoy lesion of the duodenum in a child. Case report and review of the literature. J Pediatr Surg.

